# High-resolution electric power load data of an industrial park with multiple types of buildings in China

**DOI:** 10.1038/s41597-023-02786-9

**Published:** 2023-12-06

**Authors:** Kaile Zhou, Dingding Hu, Rong Hu, Jiong Zhou

**Affiliations:** 1 School of Management, Hefei University of Technology, Hefei, 230009 China; 2 Philosophy and Social Sciences Laboratory of Data Science and Smart Society Governance, Ministry of Education, Hefei University of Technology, Hefei, 230009 China; 3 Anhui Provincial Key Laboratory of Philosophy and Social Sciences for Smart Management of Energy & Environment and Green & Low Carbon Development, Hefei University of Technology, Hefei, 230009 China; 4 Jizhong Energy Technology Services (Shanghai) Company, Shanghai, 200040 China

**Keywords:** Energy management, Electrical and electronic engineering

## Abstract

Considering the growing demand for electricity in industrial parks, understanding their electric power load patterns is critical for improving energy efficiency and ensuring the rational utilization of energy resources. However, the detailed electric power load data of various buildings in industrial parks are rarely available and accessible, which hinders the related studies. In this context, we present the electric power load data of 6 years (from January 1, 2016 to December 31, 2021) for various types of buildings in an industrial park in Suzhou, China. The data are obtained from smart meters and have various time resolutions (i.e., 5 minutes, 30 minutes, and 1 hour). This work describes the data collection, processing process, and different imputation methods. The high-resolution electric power load data can be used for various research tasks, including load prediction, load pattern recognition, anomaly detection, and demand response strategy development.

## Background & Summary

An industrial park comprises various types of buildings, and each of these buildings has its own energy consumption characteristics, especially in terms of electric power consumption. Therefore, load pattern recognition and load management of buildings are critical for the energy efficiency improvement of industrial parks^1^. Open electric power load data of industrial park can better support these efforts.

Various applications related to electric power load data are continuously being developed^2–7^. In particular, data analytics and machine learning techniques are increasingly being used in electric power load prediction and pattern recognition of buildings^8,9^. Kong *et al*. proposed a method for the anomalous electricity consumption behavior detection by extracting temporal and spatial characteristics of building electricity consumption data from real-world scenarios^10^. The anomalous detection of electricity consumption data can improve the efficiency of building electricity usage and reduce related nontechnical losses. Huebner *et al*. analyzed the correlation between electric power load and some influence factors, such as building characteristics, socio-demographics, appliance use, and self-reported behaviors, based on the sample data of more than 800 English households^11^.

Having high-quality electric power load data at the building level is crucial since it provides insights into the detailed building operation schedules^12^. A primary method to obtain electric power load data in buildings is the use of smart meters, also known as intelligent energy meters or advanced metering infrastructure (AMI)^13^. Smart meters can provide real-time consumption information, which is beneficial for understanding electricity usage patterns and identifying areas for energy saving^14,15^. With smart meters being increasingly deployed, considerable electric power load data are collected, providing new research opportunities^16^.

However, such comprehensive datasets are rarely available for research purposes or sometimes unavailable at all. Furthermore, some of the available load datasets are only available at the household level but not at the building level within an industrial park^17^. This study focuses on providing publicly available electric power load data of various buildings in an industrial park, which contributes to the regional diversification of available energy datasets globally. Specifically, we present the electric power load data of four types of buildings in an industrial park in Suzhou from 2016 to 2021, with resolutions of 5 minutes, 30 minutes, and 1 hour.

This dataset has the following unique characteristics and the potential values.

- The dataset is diverse since it includes electric power load data of different types of buildings in an industrial park. Such diversity gives rise to abundant research prospects, allowing to investigate load variation patterns in response to different building operational hours and occupant behaviors.
- The dataset has different time resolutions (i.e., 5 minutes, 30 minutes, and 1 hour). These high-resolutions allow to analyze load patterns at distinct time scales, facilitating a comprehensive understanding of load patterns in industrial parks. Furthermore, the availability of multi-resolution data is beneficial for comparing different data analysis algorithms. The uniqueness of this dataset lies not only in the data itself, but also in the efficient benchmarking of complex analytical techniques and algorithmic innovations.
- The dataset spans the period of 6 years from January 1, 2016 to December 31, 2021, which allows to capture both short-term fluctuations and long-term trends in power load. Researchers can identify seasonal patterns, track load variations over multiple years, and discern changing consumption behaviors.
- The corresponding hourly weather data has been integrated into the dataset. This integration facilitates researchers in linking load patterns with diverse weather conditions to develop and train machine learning models. Furthermore, researchers can gain an understanding of the interaction between external environmental factors and power consumption by analyzing load variations in response to weather fluctuations.

This dataset has great potential for improving the sustainability of buildings. Table 1 summarizes some of the potential applications of this dataset.

**Table 1:** Potential applications of the dataset.

Application areas	Description
Load forecasting	Prediction of load demand based on historical load demand, weather data, and relevant external factors
Load pattern recognition	Revealing unique load behavior, load variations, and other relevant characteristics by data analysis techniques
Anomaly detection	Detection of deviations from expected load behavior and further analysis of these anomalies
Demand response (DR) strategy development	Development of targeted DR strategy based on power consumption behaviors in buildings

Load forecasting refers to estimating future load demand based on historical usage patterns and external influential factors. For instance, González-Vidal *et al*. developed transferable predictive models for buildings with sharing characteristics that can improve accuracy for buildings with little data^18^. Such studies are beneficial for the improvement of building energy efficiency and the stable operation of power systems, since accurate prediction of peak load can support the implementation of peak shaving strategies^19^.

Load pattern recognition is an important way for understanding different load characteristics^20^. For example, Zhou *et al*. used a clustering algorithm to identify the power load patterns of school buildings and analyzed the power consumption in student dormitories^21^. This analytical approach supports the implementation of different energy-saving measures based on the load patterns.

Anomaly detection involves identifying deviations from expected load behavior, which can indicate faults or abnormal conditions in the power system. Cui *et al*. proposed a machine learning-based anomaly detection method that can identify network attacks using load data^22^. This application is important to reduce nontechnical loss and promote the efficient operation of power system operation^23^.

DR strategy development is another application area. Chen *et al*. evaluated the flexibility of building power demand and emphasized the importance of DR in improving energy flexibility^24^. Such research can lead users to adjust electricity using behaviors, thereby balancing electricity supply and demand in buildings.

## Methods

This section describes the methods applied for data collection and processing. Information is reported for each collected subset.

### Data collection

**Fig. 1:**
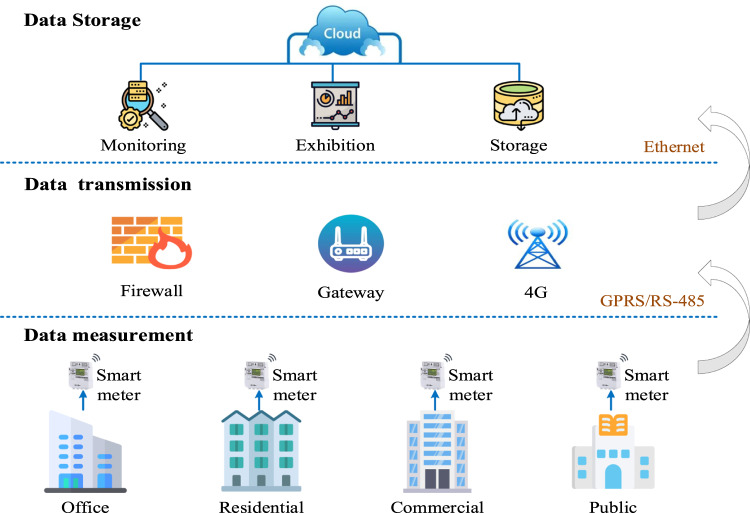
Architecture of the electric power load data collection.

The data collected originate from a smart energy management system of the industrial park in Suzhou, China. The architecture of the electric power load data collection is presented in Fig. 1, which includes data measurement, data transmission, and data storage. Firstly, smart meters measure real-time load data from the building and transmit data through RS-485/GPRS. This process simultaneously improves the security and stability of the network. The cloud platform performs monitoring, exhibition, and storage for the received data. Once the data is stored, users can access the system for data collection. Characteristics of the buildings are summarized in Table 2.

**Table 2:** Characteristics of the buildings.

Building type	Building area [m^2^]	Number of people	EUI [kWh/m^2^·year]
Office building	80,000	1,200	52.8
Residential building	107,000	3,200	18.2
Commercial building	98,000	500	1,083
Public building	24,000	700	1,004

Data retrieval was conducted on April 10, 2023, and covered a time span from January 1, 2016 to December 31, 2021. The industrial park contained various types of buildings, and the electric power load data of each building were automatically collected every 5 minutes, 30 minutes, and 1 hour by smart meters. Similarly, the weather data were automatically collected every hour by a dedicated meteorological station installed on-site.

Table 3 presents the percentage of missing values in the electric power load data for four building types over the 6 years period from 2016 to 2021. We only present the missing rate of data with a resolution of 1 hour, as the missing rates are close between data with different resolutions for the same building. Here, we need to emphasize that the higher missing rate for the public building in 2016 is due to the fact that the installation of smart meters in the public building only started in the second half of that year. There is a higher rate of missing data for residential and public buildings in 2020 and 2021, which is mainly due to the COVID-19 epidemic, which affected the buildings’ operations and the devices’ maintenance.

**Table 3:** Proportion of missing values.

Building type	Missing rate per year (%)					
	2016	2017	2018	2019	2020	2021
Office building	2.892	1.478	0.171	1.220	17.349	3.378
Residential building	3.097	1.050	5.171	3.824	32.479	33.311
Commercial building	11.452	17.551	11.130	7.021	5.316	5.719
Public building	57.399	0.913	1.256	0.765	62.261	84.087

### Data processing

Due to the potential existence of irregular behaviors and missing data in the dataset, we handled these issues throughout the data cleaning process and subsequent analyses. The proposed data contained two types of missing values. The first type is that some data are missing in a day, and the other type is that all electric power load data are empty values in a day. We handled both types of missing values in the same manner and classified them as “missing”. All requested values that were successfully retrieved from the smart energy management system (i.e., not “missing”) were initially classified as “normal”.

Multiple imputation by chained equations (MICE) is a widely used approach that transforms the problem of imputing missing data into a series of conditional imputation problems based on the assumption of conditional distributions. Specifically, in the MICE method, a variable is iteratively selected from the set of variables containing missing values as the target variable. Subsequently, the missing values of that target variable are imputed based on the known values of other variables. This process is repeated multiple times until all variables have their missing values imputed^25^. In the MICE method, the underlying correlation structure in the data is used to estimate the missing values. By repeatedly estimating the conditional distributions of the target variables in each iteration step, the MICE method gradually approximates the true distribution of the missing values and provides multiple imputed datasets as the output. These imputed datasets reflect the uncertainty of the missing values. Subsequently, analysis based on these datasets can be performed.

**Fig. 2:**
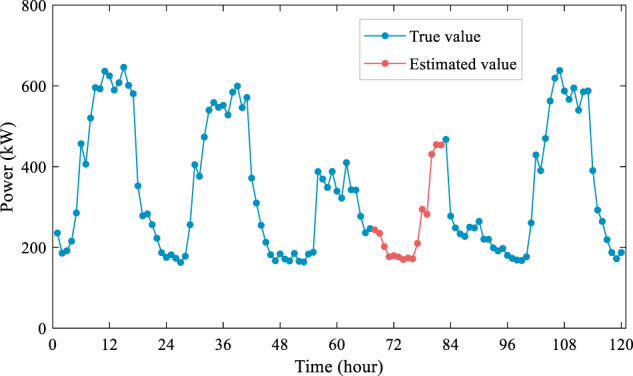
Handling missing values based on the MICE method.

The advantages of the MICE method include the ability to retain the sample size in the presence of missing data, provide reliable estimates and standard errors, and allow statistical inference and model comparison. However, the MICE method relies on certain assumptions, such as the missing data mechanism being random or conditionally random, and the imputation models accurately capturing the relevant structure of the data. Fig. 2 displays the results of supplementing the missing values of the office building on a given day using the MICE method. Other imputation methods are described in Technical Validation.

## Data Records

**Fig. 3:**
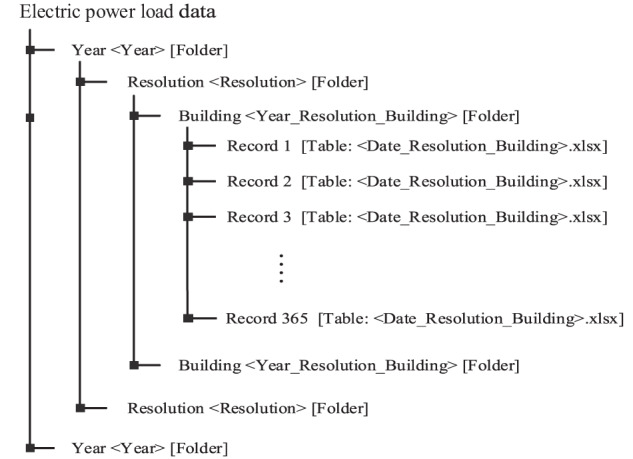
Data storage architecture.

The dataset is deposited in the Open Science Framework (OSF) data repository^26^. The electric power load data for all buildings can be made available for download through the smart energy management system of the industrial park. Timestamp is in Coordinated Unversal Time + 8 (UTC + 8), as YYYY-MM-DD hh:mm:ss. Daily data are provided in xlsx files. The year, resolution, and building type should be determined in advance before downloading data. We downloaded and sorted the dataset. The specific method to obtain data is presented in the Code availability section. Fig. 3 displays the architecture of data storage and file name format. First, we divided the entire dataset into six folders based on year, and each folder included subfiles in three resolutions: 5 minutes, 30 minutes, and 1 hour. The folder for each resolution includes the electric power load data for four types of buildings, and the name of the folder is Year_Resolution_Building. For example, the folder named “2018_1hour_Office” contains the electric power load data of office building in 1 hour resolution during the whole year of 2018, in 365 xlsx files. In the folder, the xlsx file named “20180101_1hour_Office” is the hourly electric power load data of the office building on January 1, 2018. The data in the table are in kW units. Furthermore, the date data in the table are timestamped in the local time zone of the country.

Similar to the electrical power load data, weather data is stored in the form of xlsx files. Each xlsx file contains three columns corresponding to the date, temperature, and humidity. All temperatures are in degrees Celsius. Humidity values are in percent relative humidity. The xlsx file is named using the date because there is only one resolution. For example, “20180101” is an xlsx file of hourly weather data on January 1, 2018.

## Technical Validation

### Missing value imputation

To improve the availability of dataset, different imputation methods are used to fill in the missing value. The missing value imputation process is as follows: we verify the performance of different imputation methods on a complete dataset. Then, we select the most suitable imputation method based on the evaluation metric.

**Fig. 4:**
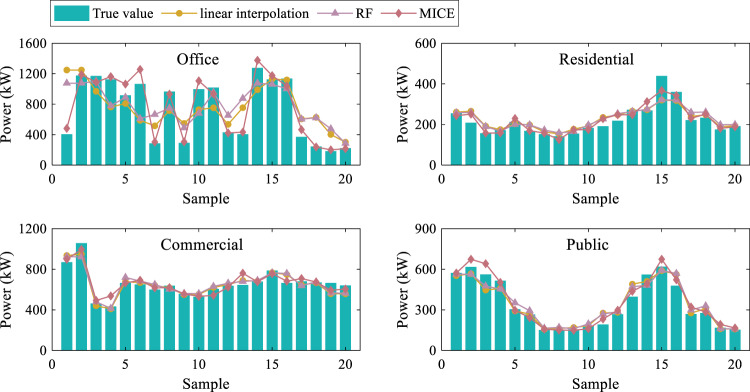
Results of different imputation methods.

First, 20 “normal” values are randomly selected from electric power load data of each building and marked as “missing”. Subsequently, different imputation methods are used for the imputation, and the estimated results are qualitatively evaluated. The selected imputation methods include linear interpolation, random forest (RF), and MICE. The code for these interpolation methods is available on our github page. The mean absolute percentage error (MAPE) serves as the evaluation metric.

1 $$MAPE=\frac{1}{N}{\sum }_{i=1}^{N}\left|\frac{{L}_{i}-{\widehat{L}}_{i}}{{L}_{i}}\right|$$

where *N* is the total number of samples, ${\widehat{L}}_{i}$ is the imputed value, and *L*_*i*_ is the actual observation value. Fig. 4 shows the estimated results based on different imputation methods. Assessment results of different imputation methods are described in Table 4.

**Table 4:** Assessment results of different imputation methods.

Building type	MAPE		
	Linear interpolation	RF	MICE
Office building	0.525	0.549	0.079
Residential building	0.098	0.111	0.082
Commercial building	0.055	0.061	0.054
Public building	0.094	0.111	0.084

For office building, linear interpolation and RF lead to bad results, showing significantly higher MAPE than MICE method. In contrast, the difference among different interpolation methods is not significant for the other three buildings, whereby RF shows slightly worse results than the other methods. Overall, it can be concluded that MICE method seems to give the best imputation results. Finally, it must be noted that since the current sample is randomly selected, the application of the aforementioned three imputation methods to other missing values in this dataset or other datasets may lead to different performance.

### Fault value diagnosis

We do not deal with the fault values, and the reasons can be summarized as follows. First, the fault values may reflect valuable information. For example, in some situations, fault or abnormal values may imply new findings on different load pattern or load consumption trends. Removing or replacing these values may result in the loss of critical information. Second, the fault values may be beneficial for constructing robust analytical model in real-world applications. Third, the definition of “fault values” is not clear. For example, if we find a very large value in the data, we cannot simply determine whether it is caused by a faulty meter or just the surge in load demand. Finally, the preservation of the raw values in the dataset contributes to anomaly detection related research.

### Correlation analysis

**Fig. 5:**
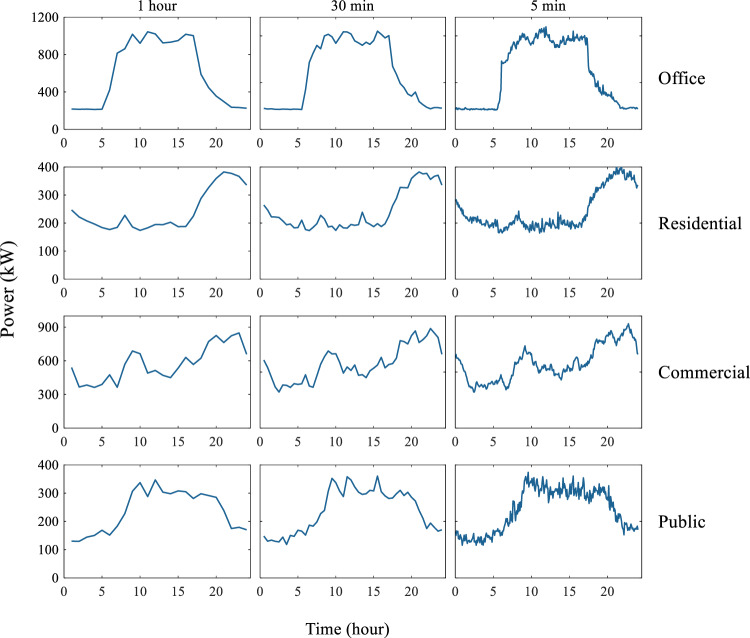
Electric power load patterns change with temporal resolution.

The first step of validation involves separating samples by constructing blocks and resampling, resulting in time steps of 5 minutes, 30 minutes, and 1 hour. Studies have indicated that higher resolution facilitates the differentiation of distinct electric power load patterns, making their characteristics more evident^27,28^. Fig. 5 illustrates power consumption patterns with different resolutions (5 minutes, 30 minutes, and 1 hour) on a representative day (March 1, 2018). The overall shape of the patterns at different resolution remains similar. Although data with 5 minutes or 30 minutes resolution provide more detailed representation of daily electric power load patterns for various building types, the 1 hour resolution data appear to be clearer. Consequently, we depicted the characteristics of the dataset in a resolution of 1 hour because it is easy to comprehend. We checked and validated the electric power load data of various types of buildings at 1 hour resolution in all subsequent steps by using data in 2018 as an example.

**Fig. 6:**
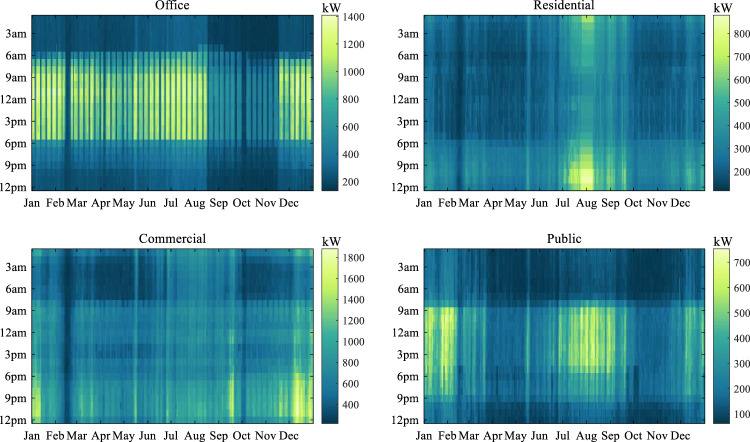
Heatmap depicting electric power load across 24 hours in each building for the year 2018.

**Fig. 7:**
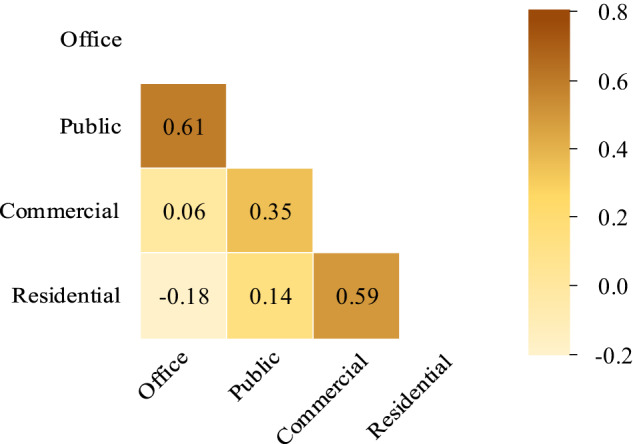
Correlations in the hourly power load of four buildings.

The 1 hour resolution electric power load of four buildings, including office, residential, commercial, and public, in 2018 are presented using heatmaps, as shown in Fig. 6. The heatmaps clearly illustrate the electric power load across 24 hours for each building in 2018. The results reveal that all buildings share a common pattern of higher power consumption during summer and winter, and lower consumption during spring and autumn. Specifically, offices exhibit a distinct peak in power consumption during daytime hours, while residences show a bimodal pattern with peaks during early morning and evening hours. Moreover, the commercial building has higher power consumption during the night hours, whereas the public building exhibits a peak in the afternoon. The power consumption of these four types of buildings is determined by the subjects and activities in the buildings. The correlations in the hourly power load of four buildings are shown in Fig. 7.

**Fig. 8:**
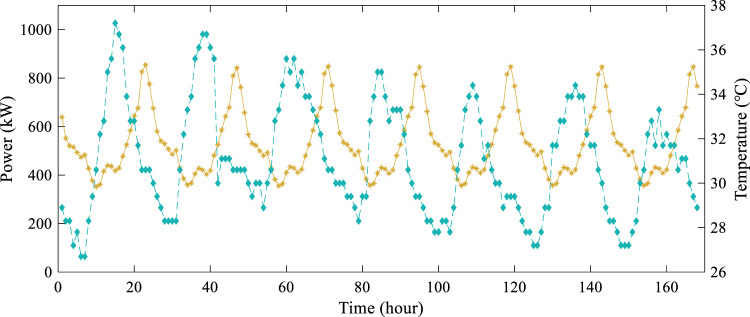
Hourly electric power load of the office building and temperature from July 25, 2018 to July 31, 2018.

It is well-known that weather factors, especially temperature, have a significant impact on electric power load. Fig. 8 shows the electric power load of the office building and temperature during a week with 1 hour resolution. It can be seen that load peaks always occur a few hours after the temperature reaches its maximum value, suggesting that temperature lag can serve as a key parameter for load forecasting.

**Fig. 9:**
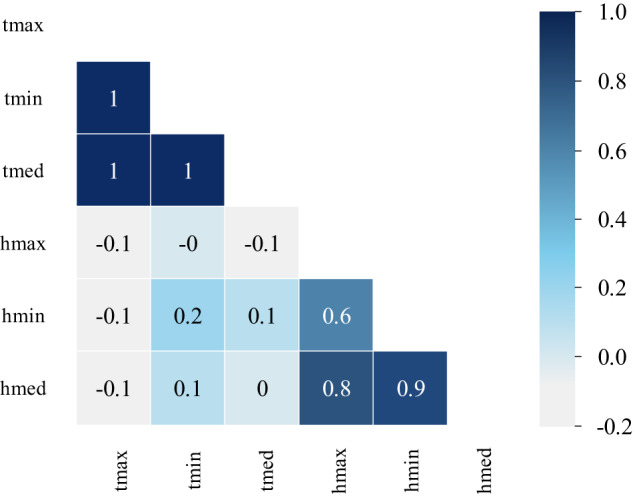
Weather correlations.

The features extracted from weather data are essential inputs for machine learning models. Considering the presence of similar or redundant input features among the original input features, we plot the correlation between the variables of the weather dataset in Fig. 9.

In Fig. 9, tmax, tmin, and tmed represent the maximum, minimum, and mean values of temperature, respectively. hmax, hmin, and hmed represent the maximum, minimum, and mean values of humidity, respectively.

Based on the above analysis, we take the load forecasting as an example to demonstrate the specific workflow of using weather data.

- Constructing dataset based on history load and weather data, and performing necessary normalization and missing values filling;
- Determining input features for the model;
- Training load forecasting model based on the dataset and selected input features;
- Predicting load based on the trained model.

### Load curve

**Fig. 10:**
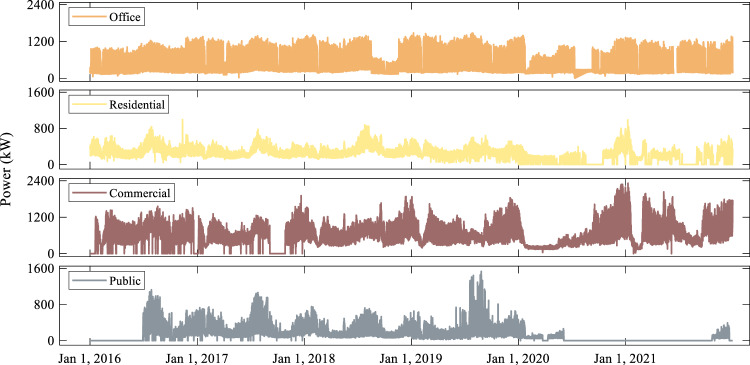
Load curve of each building from 2016 to 2021.

Fig. 10 shows the load curve for each building from 2016 to 2021. As can be seen, there are differences in the electric power load of the four buildings in different years, and the magnitude of the differences varies. The overall shape of the annual electric power load curve for office buildings is relatively similar. For the residential building, the overall power consumption level has decreased after 2019. The commercial building shows the most significant year-to-year variations, and the overall power consumption level exhibits a year-by-year increasing trend. The annual variations in the overall power consumption level of public buildings appear to be irregular.

## Usage Notes

The dataset provided in this article can be loaded into any software that supports xlsx files. To process the dataset, researchers can use programming languages such as Python, Java, MATLAB, or R. This dataset holds significant value for researchers and analysts in the energy sector, as it can be used for analysis over a 6 years period in various temporal resolutions (i.e., 5 minutes, 30 minutes, and 1 hour). The dataset can also be used for various research areas, such as load prediction, load pattern recognition, anomaly detection, and DR strategy development. Although missing values in the dataset can be added through the suggested MICE method, raw data are provided to allow researchers and related practitioners to perform their own analysis and make decisions about these data.

The smart energy management system of the industrial park can collect real-time electric power load data of each building. The dataset can be updated and the time span can be extended.

## Data Availability

The code implementation is done in the R programming language version 4.1.0 and MATLAB R2018a. The custom code used for data processing, technical validation, visualization is available on the github page (https://github.com/Industrialpark/SEMLab_HFUT-Building-Electricpowerloaddata).

## References

[1] Lei L (2023). A dynamic anomaly detection method of building energy consumption based on data mining technology. Energy.

[2] Faustine A, Pereira L, Klemenjak C (2020). Adaptive weighted recurrence graphs for appliance recognition in non-intrusive load monitoring. IEEE Trans. Smart Grid.

[3] Afzalan M, Jazizadeh F (2019). Residential loads flexibility potential for demand response using energy consumption patterns and user segments. Appl. Energy.

[4] Pereira L, Nunes N (2020). Understanding the practical issues of deploying energy monitoring and eco-feedback technology in the wild: Lesson learned from three long-term deployments. Energy Rep..

[5] Granderson J, Lin G, Harding A, Im P, Chen Y (2020). Building fault detection data to aid diagnostic algorithm creation and performance testing. Sci. Data.

[6] Rashid H, Singh P, Stankovic V, Stankovic L (2019). Can non-intrusive load monitoring be used for identifying an appliance’s anomalous behaviour?. Appl. Energy.

[7] Anand P (2021). Occupancy-based energy consumption modelling using machine learning algorithms for institutional buildings. Energy Build..

[8] Ding Y, Wang Q, Wang Z, Han S, Zhu N (2019). An occupancy-based model for building electricity consumption prediction: A case study of three campus buildings in Tianjin. Energy Build..

[9] Kim MK, Kim YS, Srebric J (2020). Predictions of electricity consumption in a campus building using occupant rates and weather elements with sensitivity analysis: Artificial neural network vs. linear regression. Sustain. Cities Soc..

[10] Kong J, Jiang W, Tian Q, Jiang M, Liu T (2023). Anomaly detection based on joint spatio-temporal learning for building electricity consumption. Appl. Energy.

[11] Huebner G, Shipworth D, Hamilton I, Chalabi Z, Oreszczyn T (2016). Understanding electricity consumption: A comparative contribution of building factors, socio-demographics, appliances, behaviours and attitudes. Appl. Energy.

[12] Yoon Y, Jung S, Im P, Gehl A (2022). Datasets of a Multizone Office Building under Different HVAC System Operation Scenarios. Sci. Data.

[13] Han D, Bai H, Wang Y, Bu F, Zhang J (2023). Day-ahead aggregated load forecasting based on household smart meter data. Energy Rep..

[14] Komatsu H, Kimura O (2023). Customer segmentation based on smart meter data analytics: Behavioral similarities with manual categorization for building types. Energy Build..

[15] Wang C, Du Y, Li H, Wallin F, Min G (2019). New methods for clustering district heating users based on consumption patterns. Appl. Energy.

[16] Thorve S (2023). High resolution synthetic residential energy use profiles for the United States. Sci. Data.

[17] Schlemminger M, Ohrdes T, Schneider E, Knoop M (2022). Dataset on electrical single-family house and heat pump load profiles in Germany. Sci. Data.

[18] González-Vidal A, Mendoza-Bernal J, Niu S, Skarmeta AF, Song H (2022). A Transfer Learning Framework for predictive energy-related scenarios in Smart Buildings. IEEE Trans. Ind. Appl..

[19] Amara-Ouali Y, Fasiolo M, Goude Y, Yan H (2023). Daily peak electrical load forecasting with a multi-resolution approach. Int. J. Forecast..

[20] do Carmo CMR, Christensen TH (2016). Cluster analysis of residential heat load profiles and the role of technical and household characteristics. Energy Build..

[21] Zhou Y, Sun L, Hu X, Ma L (2021). Clustering and statistical analyses of electricity consumption for university dormitories: A case study from China. Energy Build..

[22] Cui M, Wang J, Yue M (2019). Machine learning-based anomaly detection for load forecasting under cyberattacks. IEEE Trans. Smart Grid.

[23] Hosseini SS, Agbossou K, Kelouwani S, Cardenas A, Henao N (2020). A practical approach to residential appliances on-line anomaly detection: A case study of standard and smart refrigerators. IEEE Access.

[24] Chen Y, Xu P, Gu J, Schmidt F, Li W (2018). Measures to improve energy demand flexibility in buildings for demand response (DR): A review. Energy Build..

[25] Ruggles TH, Farnham DJ, Tong D, Caldeira K (2020). Developing reliable hourly electricity demand data through screening and imputation. Sci. Data.

[26] Zhou K, Hu D, Hu R, Zhou J (2023). Open Science Framework.

[27] Carrie Armel K, Gupta A, Shrimali G, Albert A (2013). Is disaggregation the holy grail of energy efficiency? The case of electricity. Energy Policy.

[28] Pipattanasomporn M (2020). CU-BEMS, smart building electricity consumption and indoor environmental sensor datasets. Sci. Data.

